# Impact of streptozotocin-induced diabetes on experimental masseter pain in rats

**DOI:** 10.1590/1807-3107bor-2024.vol38.0073

**Published:** 2024-08-05

**Authors:** Yuri Martins COSTA, Clarissa Carolina Fernandes HERCULIANI, Flávia Fonseca Carvalho SOARES, Michelle de Campos Soriani AZEVEDO, Paulo César Rodrigues CONTI, Thiago José DIONÍSIO, Gabriela de Moraes OLIVEIRA, Flávio Augusto Cardoso de FARIA, Carlos Ferreira SANTOS, Gustavo Pompermaier GARLET, Leonardo Rigoldi BONJARDIM

**Affiliations:** (a) Universidade Estadual de Campinas – Unicamp, Piracicaba Dental School, Department of Biosciences, Piracicaba, SP, Brazil.; (b) Universidade de São Paulo – USP, Bauru School of Dentistry, Department of Biological Sciences, Bauru, São Paulo, SP, Brazil.; (c) Universidade de São Paulo – USP, Bauru School of Dentistry, Department of Prosthodontics, Bauru, SP, Brazil.; (d) Universidade de São Paulo – USP, Hospital for Rehabilitation of Craniofacial Anomalies, Bauru, SP, Brazil.

**Keywords:** Diabetes Mellitus, Diabetic Neuropathies, Nociception, Masseter Muscle, Pain

## Abstract

This study aimed to assess the influence of streptozotocin (STZ)-induced diabetes on the nociceptive behavior evoked by the injection of hypertonic saline (HS) into the masseter muscle of rats. Forty male rats were equally divided into four groups: a) isotonic saline control, which received 0.9% isotonic saline (IS), (Ctrl-IS); b) hypertonic saline control, which received 5% HS (Ctrl-HS); c) STZ-induced diabetic, which received IS, (STZ-IS); d) STZ-induced diabetic, which received HS (STZ-HS). Experimental diabetes was induced by a single intraperitoneal injection of STZ at dose of 60 mg/kg dissolved in 0.1 M citrate buffer, and 100 μL of HS or IS were injected into the left masseter to measure the nociceptive behavior. Later on, muscle RNA was extracted to measure the relative expression of the following cytokines: cyclooxygenase-2 (COX-2), tumor necrosis factor (TNF-α), and interleukins (IL)-1β, -2, -6, and -10. One-way analysis of variance (ANOVA) was applied to the data (p < 0.050). We observed a main effect of group on the nociceptive response (ANOVA: F = 11.60, p < 0.001), where the Ctrl-HS group presented the highest response (p < 0.001). However, nociceptive response was similar among the Ctrl-IS, STZ-IS, and STZ-HS group (p > 0.050). In addition, the highest relative gene expression of TNF-α and IL-6 was found in the masseter of control rats following experimental muscle pain (p < 0.050). In conclusion, the loss of somatosensory function can be observed in deep orofacial tissues of STZ-induced diabetic rats.

## Introduction

Diabetes mellitus (DM) is a chronic metabolic disease characterized by hyperglycemia, which results from failure of insulin secretion or action. The worldwide population prevalence is estimated at 8.3%.^1^

Diabetic peripheral neuropathy (DPN) is a common complication of DM that is associated with somatosensory alterations. DPN can affect up to 50% of diabetic patients and is one of the main causes of morbidity and mortality.^2^ Patients with DPN may experience signs and symptoms of somatosensory amplification, e.g., spontaneous pain, hyperalgesia to mechanical and thermal stimuli and mechanical allodynia, but also signs and symptoms of sensory loss, e.g. numbness and mechanical and thermal hypoesthesia.^3,4^ These somatosensory phenotypes are more pronounced in the distal parts of superficial tissues, especially in the lower extremities.^2-4^

Nevertheless, the etiopathogenesis mechanisms of DPN are not well established yet. Currently, prevention, diagnostic, and therapeutic strategies for DPN are deficient due to the various forms of pathogenesis of systemic and cellular disorders in glucose and lipid metabolism.^5-7^ These abnormalities lead to the activation of complex biochemical pathways, including increased tumor necrosis factor (TNF-α), interleukin (IL)-1β, IL-2, IL-6, IL-10, and cyclooxygenase-2 (COX-2).^6,7^ In addition, genes involved in neuronal damage, cyclooxygenase-2 activation, and low-grade inflammation are activated.^7^ Recently, many experimental and clinical studies have shown an important role of long-term low-grade inflammation in DPN pathogenesis, suggesting that inflammation is the common denominator of nerve damage and pain in diabetes.^7-10^ It seems that a systemic inflammatory process occurs in peripheral neuropathy, particularly if the peripheral neuropathy is associated with neuropathic pain.^9^

The administration of a single dose of streptozotocin (STZ), an antibiotic extracted from Streptomyces Achromogenes, which selectively destroyed β-cells in pancreatic islets, is associated with early neuropathic phenotypes in animal experiments.^11-13^ For instance, signs of somatosensory amplification such as orofacial thermal hyperalgesia and signs of somatosensory loss such as lower limb mechanical hypoalgesia have been identified in rodents following a single dose of STZ.^14,15^ These investigations have contributed to better elucidate neuropathic pain mechanisms related to diabetes.^11^ Nonetheless, potential consequences of DPN for the sensitivity of deep tissues, e.g. skeletal muscles, are not sufficiently reported. Although there is evidence that STZ-induced diabetes can modulate the activity of the adenyl cyclase system in skeletal muscles of rats,^16^ behavior phenotyping associated with experimental muscle pain has not been investigated so far.

Based on the above, the primary aim of the present study was to assess the influence of STZ-induced diabetes on the nociceptive behavioral response evoked by an intramuscular injection of hypertonic saline (HS) into the masseter muscle of rats. We hypothesized that the nociceptive behavioral responses would differ between STZ-induced diabetes rats and control rats without diabetes. In addition, we investigated the influence of STZ-induced diabetes and HS injection on the gene expression of the following inflammatory biomarkers: TNF-α, interleukins (IL)-1β, -2, -6 and -10, and COX-2.

## Methods

### Animals

The present study was conducted in 40 male Wistar rats (200-250 g) maintained under controlled conditions of temperature (23 ± 2°C), humidity and light-dark cycle (12 h), and with access to food and water “ad libitum”. The experiments were conducted during the light phase of the circadian cycle, between 8 and 17 hours. The examiner initially manipulated each animal for a period of 3 days before the beginning of the experiments. Experimental protocols were approved by the Ethics Committee on Animal Education and Research, Bauru School of Dentistry, University of São Paulo (#005/2015) and conducted in accordance with accepted standards of humane animal care, as outlined in the Ethical Guidelines.^17^ This study complies with ARRIVE guidelines, UK Animals (Scientific Procedures) Act (1986), EU Directive 2010/63/EU for experiments on animals, National Institutes of Health Guidance for the Care and Use of Laboratory Animals (NIH Publications No. 8023), Federal Law No. 11,794/08 (Arouca Law), and the Brazilian Practice Guideline for the Care and Use of Animals for Scientific and Teaching Purposes (DBPA).

The rats were equally divided into four independent groups: 1) normoglycemic control rats that received 0.9% isotonic saline (IS) (n = 10, Ctrl-IS); 2) normoglycemic control rats that received hypertonic saline 5% (HS), (n = 10, Ctrl-HS); 3) STZ-induced diabetic rats that received IS (n = 10, STZ-IS); 4) STZ-induced diabetic rats that received HS (n = 10, STZ-HS). One hour after the beginning of the orofacial nociception tests, the animals were euthanized by an intraperitoneal overdose injection of sodium thiopental (Thiopentax^®^, Cristália-Química e Farmacêutica, São Paulo, Brazil) (150 mg/kg i.p.), associated with an intramuscular injection of the anesthetic lidocaine hydrochloride (Xylestesin^®^, Cristália-Química e Farmacêutica, São Paulo, Brazil) (10 mg/kg i.m.).

### Induction of diabetes

Twenty Wistar male rats were weighed to calculate the amount of STZ (Streptozotocin - Sigma-Aldrich Co. LLC.) to be injected. Experimental diabetes was induced by a single intraperitoneal injection of STZ at dose of 60 mg/kg dissolved in 0.1 M citrate buffer, pH 4.5.^18^ Hyperglycemia was confirmed 72 h after STZ injection in a peripheral blood sample obtained from the animal’s tail, using glucometer One Touch Ultra (One Touch^®^ - Johnson & Johnson, Medical Devices & Diagnostic Group, São José dos Campos, Brazil). Blood glucose level above 250 mg/dL was required to be considered diabetic and be included in the study.^19^

### Nociceptive behavioral test

Fourteen days after the STZ-induced diabetes, orofacial nociceptive behavior was assessed in rats lightly anesthetized with thiopental sodium 40 mg/kg, i.p. (Thiopentax^®^, Cristalia - Chemicals and Pharmaceuticals, São Paulo, Brazil). The level of “light” anesthesia was determined by providing a noxious pinch to the tail or hind paw with serrated forceps. Animals typically respond to noxious tail stimulation with an abdominal contraction and a withdrawal reflex of the hind paw within 30 minutes after the initial anesthesia.^20^ Thus, the experiments continued only after the rats presented clear reflex responses for each noxious stimulus, as previously described.^20^ Therefore, with the rats lightly anesthetized, but displaying reflex responses, 100 μL of HS or IS was injected into the mid-region of the left masseter muscle, at a depth of 5 mm.^20^ A single examiner quantified the nociceptive behavioral response, which was determined by counting the numbers shaking responses of the hind paw for a period of two minutes.^20^

### Analysis of cytokine gene expression through quantitative polymerase chain reaction (qPCR)

A 1-cm^2^ fragment of the left masseter muscle was obtained one hour after the nociceptive behavioral test, and RNA was extracted using RNeasy Mini Kit (Qiagen^®^, Germany) for further analysis of cytokines expression through qPCR. The qPCR experiment was conducted according to the manufacturer’s guidelines (Applied Biosystems, USA) using specific probes for each cytokine. For the experiment, the following assays from Applied Biosystems were used: COX-2 (#Rn01483828_m1), TNF-α (# Rn01525859_g1), IL-1β (# Rn00580432_m1), IL-2 (#Rn00587673_m1), IL-6 (#Rn01410330_m1), and IL-10 (# Rn00563409_m1). The experiment was conducted in a plate of 384 wells under the following cycling conditions: initial temperature of 95°C for 10 minutes for activation of the Taq polymerase, followed by 45 cycles of 95°C for 15 seconds and 60°C for 1 minute. Negative control experiments without cDNA were also performed. Calculations for determining the relative levels of gene expression were made from triplicate measurements of the target gene normalized to β-actin, using the 2-ΔΔct method.

### Statistical Analysis

Data from the nociceptive behavioral response and relative cytokine expression were assessed for normal distribution using the Kolmogorov-Smirnov test, and a log_10_ transformation was performed when the test results were significant considering an alpha level of 5% (p < 0.050). The following variables were log_10_ transformed: COX-2, IL-1β, and IL-6. Data were reported as means and standard deviation (SD).

One-way analysis of variance (ANOVA) was computed to assess mean differences among the groups regarding the nociceptive behavioral response and relative cytokine expression. When appropriate, post-hoc analyses were performed using Tukey’s Honestly Statistical Difference (HSD). A prior planned Bonferroni correction lowered the significance level to 2.5% (p = 0.025) as the cut-off point to establish the statistical significance adjusted for multiple comparisons. The nociceptive response (main outcome) was considered one family of comparison and cytokines were regarded as another family (secondary outcomes). Therefore, the family-wise error rate was established considering 2 multiple comparisons and, according to the Bonferroni formula (0.050 / k, where k = number of comparisons), an alpha level of p = 0.025 was set up.

## Results

### Nociceptive behavioral response

**Figure 1:**
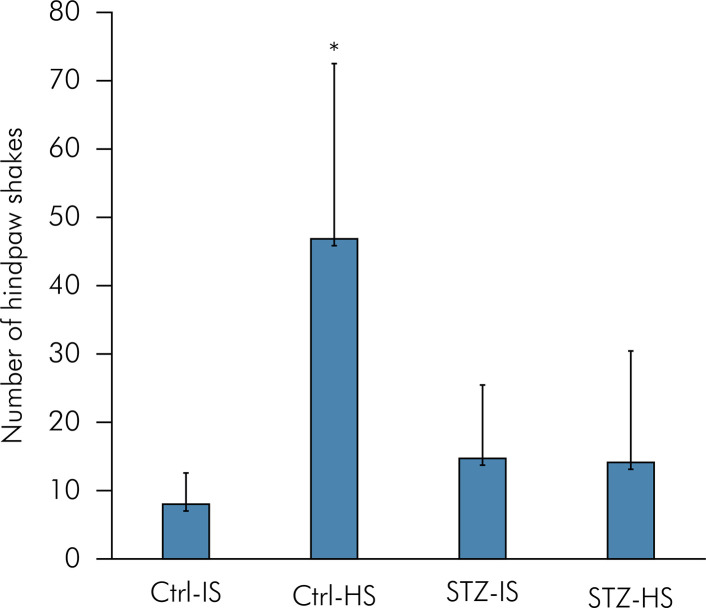
Mean number of hind paw shakes (nociceptive behavioral response). Ctrl-IS = normoglycemic control rats which received isotonic saline 0.9% (n=10). STZ-IS = streptozotocin-induced diabetic rats which received isotonic saline 0.9% (n=10). Ctrl-HS = normoglycemic control rats which received hypertonic saline 5% (n=10). STZ-HS = streptozotocin-induced diabetic rats which received hypertonic saline 5% (n=10). Error-bars indicate standard deviation (SD) of the mean. *p<0.050 compared to the other groups.

There was a main effect of group on the nociceptive response (ANOVA: F = 11.60, p < 0.001, partial η2 = 0.49), where the Ctrl-HS group presented the highest number of hind paw shaking behavior (Tukey: p < 0.001) (Figure 1). In addition, the nociceptive response was similar among the Ctrl-IS, STZ-IS, and STZ-HS groups (Tukey: p > 0.050).

### Cytokine relative gene expression

**Figure 2:**
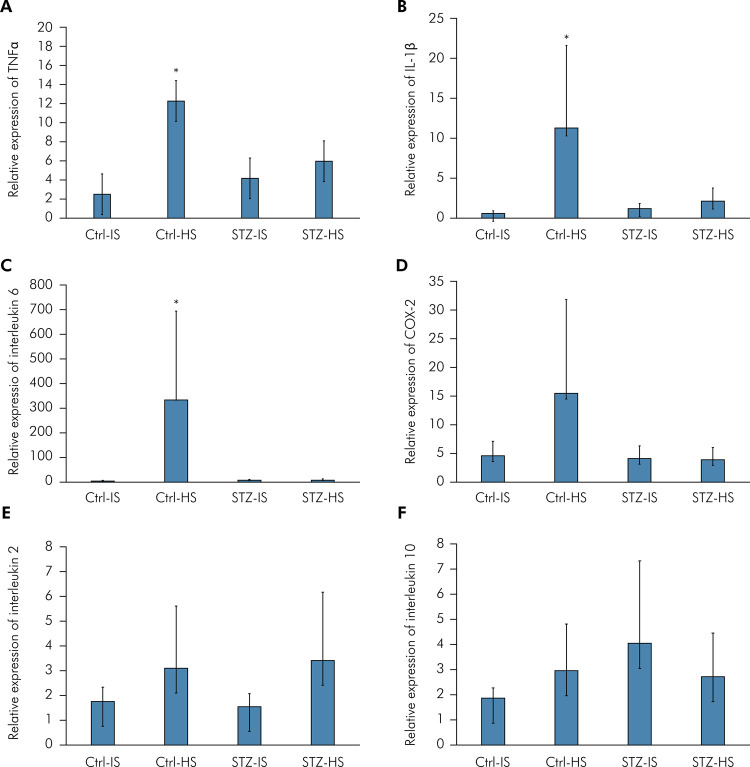
Relative expression of tumor necrosis factor (TNF-α) in the left masseter. Ctrl-IS = normoglycemic control rats which received isotonic saline 0.9% (n = 10). STZ-IS = streptozotocin-induced diabetic rats which received isotonic saline 0.9% (n = 10). Ctrl-HS = normoglycemic control rats which received hypertonic saline 5% (n = 10). STZ-HS = streptozotocin-induced diabetic rats which received hypertonic saline 5% (n = 10). Error-bars indicate standard deviation (SD) of the mean.

There was a main effect of group on gene expression of the following cytokines (Figure 2): TNF-α (Figure 2A) (ANOVA: F = 8.56, p = 0.001, partial η^2^ = 0.61), where the masseter of Ctrl-HS group presented the highest expression (Tukey: p < 0.034); IL-1β (Figure 2B) (ANOVA: F = 7.36, p = 0.002, partial η^2^ = 0.58), where the masseter of Ctrl-HS group presented a higher expression when compared to the Ctrl-IS (Tukey: p = 0.002), and STZ-IS masseter (Tukey: p = 0.015); IL-6 (Figure 2C) (ANOVA: F = 18.56, p < 0.001, partial η^2^ = 0.77), where the masseter of Ctrl-HS group presented the highest expression (Tukey: p < 0.001). In addition, gene expression of COX-2, IL-2, and IL-10 were similar among the groups (Tukey: p > 0.050) (Figures 2D, 2E, and 2F).

*p < 0.050 compared to the other groups.

Error-bars indicate standard deviation (SD) of the mean.

## Discussion

The present study aimed primarily to assess the behavioral phenotyping evoked by intramuscular injection of HS into the masseter muscle of STZ-induced diabetic rats, but also the relative expression of pro and anti-inflammatory cytokines. The main findings were: a) the impact of experimental muscle pain on the nociceptive behavioral response was significantly lower in STZ-induced diabetic rats compared to normoglycemic controls; b) normoglycemic control rats had the highest relative expression of TNF-α and IL-6 genes in the masseter following experimental muscle pain.

HS has been adequately used for induction of orofacial experimental muscle pain both in animal and human studies.^6,21^ Intramuscular injection of HS into the masseter muscle triggers trigeminal spinal nucleus neurons, which process the information from the afferent fibers of the masticatory muscle, resulting in a measurable and stereotyped nociceptive behavioral response.^20,22^ Our results demonstrated that HS evoked a significantly high nociceptive response only in the normoglycemic control rats, which indicates chemical hypoalgesia in diabetic rats following a single dose of STZ. So far, sensory loss has been reported to occur mainly in superficial tissues of STZ-induced diabetic rodents.^12^ Our results indicated that the neuropathic consequences of diabetes could also affect the afferent fibers of deep tissues, probably through the same underlying mechanisms associated with mechanical and thermal hypoalgesia, i.e. vascular and metabolic alterations from the hyperglycemia levels can cause axonal degeneration and nerve fiber loss.^2,4^ This sensory deprivation might impact orofacial motor function, as sensory feedback is important for appropriate motor activity, in particular rhythmic movements such as mastication.^23,24^ However, further investigations are required to investigate the relationship between orofacial pain and jaw function in DPN animal models.

In the present study, the expression of pro- and anti-inflammatory cytokines in the masseter muscle was also investigated 1 hour after the induction of nociception by HS and 14 days after the induction of experimental diabetes. The normoglycemic control rats injected with HS presented the highest relative expression of TNF-α and IL-6 mRNA. Likewise, this group had higher IL-1β expression compared to the normoglycemic control and STZ-induced diabetic rats injected with IS. The high level of mRNA expression in normoglycemic control rats in contrast with STZ-induced diabetic rats is probably the result of neurogenic inflammation that can lead to the antidromic release of substance P and calcitonin gene-related peptide (CGRP).^25,26^ These neuropeptides might have activated resident cells, especially resident macrophages, which in turn enhanced the expression of inflammatory cytokines.^27^ Considering that neurogenic inflammation depends on appropriate encoding and transmission of nociceptive stimuli,^25^ the absence of high expression of the above cytokines in STZ-induced diabetic rats could be considered a consequence of axonal degradation and nerve fiber loss, which might also be related to the observed hypoalgesic response. Such behavioral and biochemical outcomes could correspond to the sensory loss profile of neuropathic pain.^28,29^ Nonetheless, considering that we did not assess nerve structural changes, this interpretation should be considered with caution.

This study has some important limitations that need to be addressed: a) we only assessed early effects of STZ-induced diabetes; b) we did not assess superficial tissue sensitivity, so that a correlation of sensory changes between different tissues could not be verified; c) sensory changes and cytokine expressions were not assessed over time, which is important for a comprehensive understanding of the neurophysiological consequences of STZ-induced diabetes.

## Conclusion

Loss of somatosensory function was observed in deep orofacial tissues of STZ-induced diabetic rats. Therefore, musculoskeletal sensory assessment should be encouraged to better elucidate the neuropathic consequences of diabetes in animal experiments.
